# The Impact of Adipose Tissue–Derived miRNAs in Metabolic Syndrome, Obesity, and Cancer

**DOI:** 10.3389/fendo.2020.563816

**Published:** 2020-10-06

**Authors:** Gabriella Simões Heyn, Luís Henrique Corrêa, Kelly Grace Magalhães

**Affiliations:** Laboratory of Immunology and Inflammation, Department of Cell Biology, University of Brasilia, Brasilia, Brazil

**Keywords:** **** miRNA, adipose tissue, obesity, cancer, metabolic syndrome

## Abstract

Obesity is a multifactorial and complex condition that is characterized by abnormal and excessive white adipose tissue accumulation, which can lead to the development of metabolic diseases, such as type 2 diabetes mellitus, nonalcoholic fatty liver disease, cardiovascular diseases, and several types of cancer. Obesity is characterized by excessive adipose tissue accumulation and associated with alterations in immunity, displaying a chronic low-grade inflammation profile. Adipose tissue is a dynamic and complex endocrine organ composed not only by adipocytes, but several immunological cells, which can secrete hormones, cytokines and many other factors capable of regulating metabolic homeostasis and several critical biological pathways. Remarkably, adipose tissue is a major source of circulating microRNAs (miRNAs), recently described as a novel form of adipokines. Several adipose tissue–derived miRNAs are deeply associated with adipocytes differentiation and have been identified with an essential role in obesity-associated inflammation, insulin resistance, and tumor microenvironment. During obesity, adipose tissue can completely change the profile of the secreted miRNAs, influencing circulating miRNAs and impacting the development of different pathological conditions, such as obesity, metabolic syndrome, and cancer. In this review, we discuss how miRNAs can act as epigenetic regulators affecting adipogenesis, adipocyte differentiation, lipid metabolism, browning of the white adipose tissue, glucose homeostasis, and insulin resistance, impacting deeply obesity and metabolic diseases. Moreover, we characterize how miRNAs can often act as oncogenic and tumor suppressor molecules, significantly modulating cancer establishment and progression. Furthermore, we highlight in this manuscript how adipose tissue–derived miRNAs can function as important new therapeutic targets.

## Introduction

The worldwide statistics of overweight and obesity are considered a threat and contribute to a set of risk factors that enhances the development of several other diseases, such as type 2 diabetes, cardiovascular diseases, and cancer (1). Obesity is considered a low-grade chronic inflammation disease, which can lead to increased insulin resistance, altered preadipocyte differentiation, pre-existing adipocyte hypertrophy, macrophages infiltration and polarization, and the release of inflammatory cytokines (2).

Obesity is characterized by a dysfunction in adipose tissue homeostasis, with triglycerides accumulation and prominent adipocyte hypertrophy, resulting in hypoxia, adipocyte cell death, and free fatty acids released. There is also an intense inflammatory profile in adipose tissue from obese people due to the excessive macrophages and other immune cells infiltration to this tissue (3), which also contribute to the adipocyte cell death and free fatty acids release. Both adipocytes and immune cells are relevant sources of cytokines and chemokines secretion in adipose tissue. During obesity, the adipose tissue increases in mass and quantity, with a significant change in its metabolic and immunological profile, followed by adipocyte lytic cell death. This adipocyte death triggers free fatty acids and several metabolites release, thus pivoting the immune repertoire toward a type 1 (pro-inflammatory) state and establishing a microenvironment that supports oxidative stress, free radicals generation associated with DNA damage and mutations. These parameters can directly contribute to turning this microenvironment into a pro-carcinogenic model. Moreover, an increased free fatty acids release in adipose tissue during obesity can provide a direct faster source of energy for cancer cells, also remodeling the immune cell landscape and facilitating tumor establishment and development (4, 5). In addition, several inflammatory genes upregulation in immune cells can drive the inflammatory response itself in adipose tissue, contributing to the insulin resistance and glucose intolerance (6), critical factors associated with metabolic syndrome. Therefore, those metabolic and inflammatory changes in adipose tissue can disrupt physiological homeostasis both within local tissues and systemically, impacting directly the obesity development, as well as the initiation and progression of cancer and metabolic syndrome, creating positive feedback loops among them. Metabolic syndrome is defined as a cluster of metabolic disorders components, including obesity, cardiometabolic alterations, associated with other diseases such as type 2 diabetes, hypertension, and dyslipidemia with dysregulated levels of non-esterified fatty acids (NEFA) and decreased levels of high-density lipoprotein (HDL) (7). Metabolic syndrome leads to a higher secretion of inflammatory mediators, such as leptin, tumor necrosis factor alpha (TNF-α), monocyte chemoattractant protein-1 (MCP-1), resistin, and interleukin 6 (IL-6) (8). This prevalent inflammatory profile described in metabolic syndrome does not result from tissue injury, infection, or an autoimmune response, but instead, a meta-inflammation (9). Meta-inflammation is a chronic state of inflammation characterized by dysregulation of proinflammatory cytokines and chemokines, and mediated by macrophages (10). Recent studies have highlighted the importance of adipose tissue resident macrophages as a principal source for this inflammatory signature supporting meta-inflammation and miRNAs are key molecules in these signaling pathways (11).

## Adipose Tissue and Macrophages

Adipose tissue can be classified into white, brown, beige and pink adipose tissues, which display different regulatory, morphological and functional characteristics of their adipocyte and immune cells (12). White adipose tissue (WAT) acts as a reservoir for energy storage and secretes paracrine factors that regulate other metabolic tissues. WAT is significantly expanded during obesity triggering several harmful effects, including inflammation, altered adipokines secretion, fibrosis, hypoxia, and mitochondrial dysfunction (13). Brown adipocytes precursors can transdifferentiate into white or beige, as wells as precursors of white adipocytes can differentiate into brown and beige (14). The brown adipose tissue (BAT) is related to a higher thermogenic activity, supporting non-shivering thermogenesis, and recently identified and characterized by proteomics-based analysis, secreted factors from BAT, known as batokines, targeting immune cells, regulating glucose homeostasis and insulin sensitivity, angiogenesis, and neurite outgrowth (15, 16). Recent studies have shown the role of miRNA in adipocyte development, metabolic functions, proliferation, and differentiation (17). The beige adipose tissue is an intermediate phenotype of WAT to BAT, which when stimulated by cold exposure or β-3 adrenergic agonists may lead to increased BAT markers expression, such as the uncoupling protein 1 (UCP-1), PGC-1α, PGC-1β, CIDEA, PPARγ, and PRDM16 (18). A forth type of adipose tissue, is the pink adipose tissue mainly composed by pink adipocytes (19, 20), which are milk-secreting alveolar cells that can arise from transdifferentiation of white adipocytes during pregnancy and lactation.

Macrophages can function as key sentinel orchestrators of the immune activity in adipose tissues. Adipose tissue resident macrophages have an important role mediating meta-inflammation, secreting inflammatory mediators such as chemokines, activating the recruitment of more immune cells to the adipose tissues (21, 22). Among all immune cells within the adipose tissue, macrophages contributed predominantly to the activation of inflammatory pathways within adipose tissues during the establishment of obesity and other metabolic diseases (23).

The infiltration of macrophages in adipose tissue, surrounding dead adipocytes can form the crown-like structures (CLSs), which are directly related to increased tissue inflammation and release of free fatty acids and other pro-inflammatory molecules. In obesity, there is an exacerbated presence of CLS in WAT (24). It has been described that CLS can promote the survival and proliferation of breast cancer in patients with obesity, influencing the tumor microenvironment (25).

The exposure of adipose tissue macrophages to different pro- or anti-inflammatory cytokines can lead to two polarization profiles, M1 (pro-inflammatory) and M2 (anti-inflammatory), respectively (26). The M1 profile is induced by interferon gamma (IFN-γ), microbial products such as LPS, and tumor necrosis factor alpha (TNFα). It is characterized by the release of increased levels of reactive oxygen species, interleukins IL-12, IL-10, and IL-23 (27). The M2 phenotype is triggered by glucocorticoid hormones and interleukins IL-4, IL-13, IL-10, and increased expression of arginase 1, also related to cell growth and proliferation (28). Lumen and colleagues analyzed white adipose tissue macrophages from lean and obese mice, showing that the M1 macrophages were prevalent in adipose tissue from obese mice. Moreover, in adipose tissue from lean mice, macrophages mostly polarized to M2 profile and presented a higher expression of anti-inflammatory proteins, such as arginase (29).

**Figure 1:**
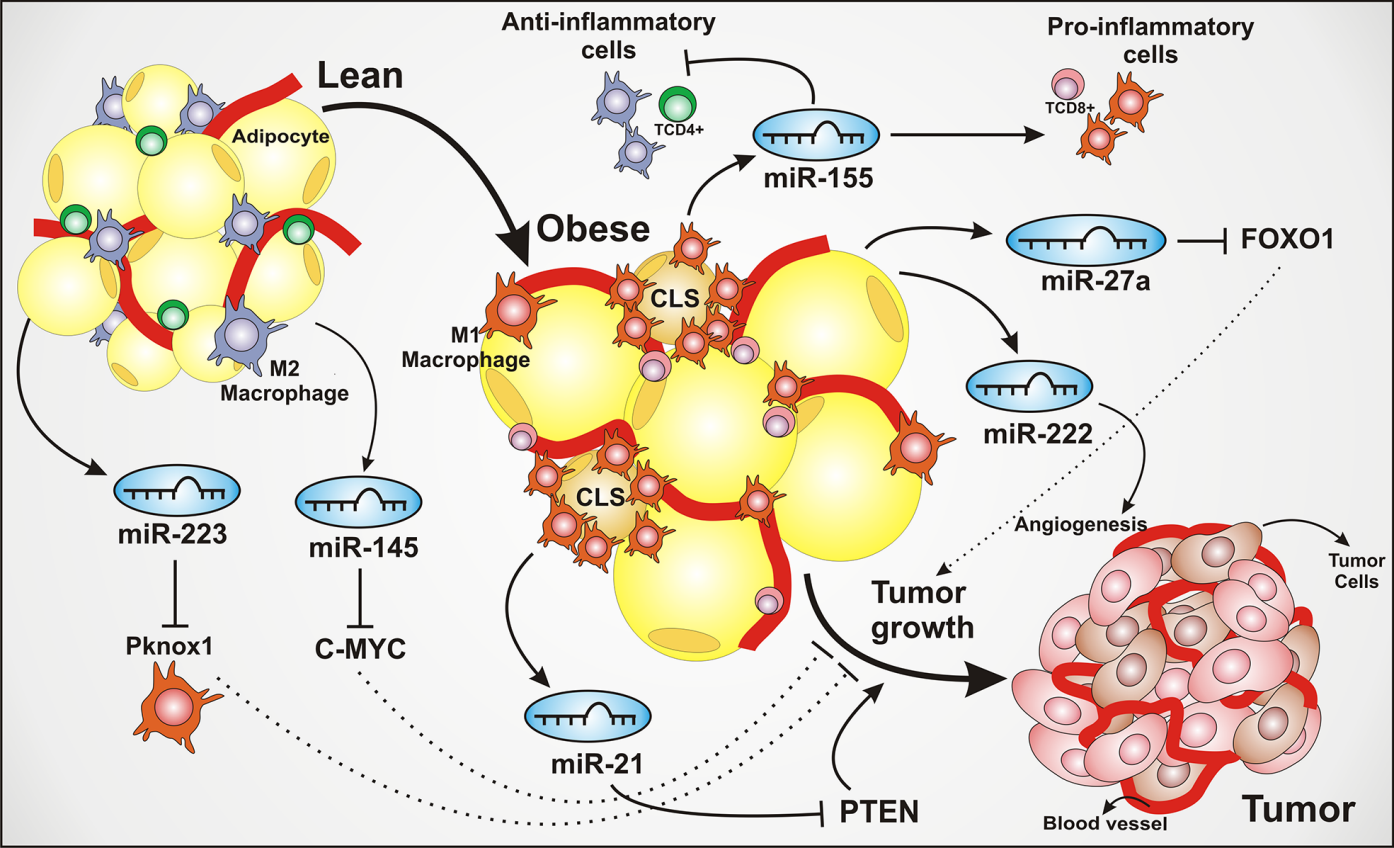
Adipose tissue–derived miRNAs modulate inflammation and tumor microenvironment. The adipose tissue produces and secretes several miRNAs that dynamically regulate the tissue homeostasis. These miRNAs can be secreted by both adipose tissue and macrophages, playing a crucial role impacting on inflammatory pathways, angiogenesis, cell growth, cell proliferation, and contributing to tumor development. The lean adipose tissue is characterized by an infiltrate of M2 polarized macrophages and TCD4+ lymphocytes. The secretion of miR-223, suppressing PBX/Knotted 1 Homeobox 1 (Pknox1), is shown to inhibit the activation of pro-inflammatory M1 macrophages, leading to an anti-inflammatory environment. The miR-145 exerts a tumor suppressor role, associated to the inhibition of oncogenic transcriptional factor C-MYC. The transition from lean to obese adipose tissue is characterized by the adipocytes hypertrophy, an increase of blood vessels and pro-inflammatory cell recruitment and polarizing as a common feature of obesity. Regarding the inflammatory recruitment, the infiltration of pro-inflammatory macrophages around the dying adipocytes is strongly present, forming structures known as CLS (crown-like structures), associated with the secretion of multiple inflammatory factors and considered an aggressiveness marker in breast cancer. The miR-21 is depicted being secreted by adipose tissue macrophages, also known as an oncomiR or oncogenic, through PTEN silencing, thus leading to the activation of tumor growth and proliferation pathways. The miR-155 contributes to the activation of the inflammatory response. The miR-222 is associated with angiogenesis, one of the tumor development hallmarks. The oncogenic miR-27a can lead to the inhibition of Forkhead box transcription factor O1 (FOXO1), an antitumoral multifunctional transcription factor, regulating genes linked to cell cycle arrest and apoptosis.

Both adipocytes and macrophages within the adipose tissue can secrete miRNAs, developing an important role between adipocytes and macrophages communication, as well as impacting on macrophages polarization and the inflammatory status of adipose tissue, as summarized in **Figure 1**. The polarization of macrophages to M1 or M2 phenotypes can be also regulated by miRNAs. Upregulation of miRNA-181a, miR-155-5p, and miR-451 is related to M1 polarization, contributing to the inflammatory status of adipose tissue (30). Adipose tissue macrophages could also regulate obesity-induced inflammation by downregulating miR-30 in high fat diet-induced obese mice, promoting the Notch1 signaling and M1 phenotype switch (31). Increased release of miR-155 by adipose tissue macrophages in obese mice is associated with insulin resistance and PPARγ modulation (32). Moreover, analysis from adipose tissue from knockout mice lacking miRNA-181, showed an increased expression of genes associated with M2 macrophages polarization as Ym1, Realm, and Arg1, while the genes related to pro-inflammatory M1 macrophage polarization were reduced (33). Therefore, miRNAs are key players in the adipocytes and immune cells crosstalk in adipose tissue, contributing significantly to the inflammatory status of this microenvironment.

## The Biogenesis of miRNAs

The miRNAs are small non-coding RNAs consisting of 19-23 nucleotides that regulate several biological processes mostly through post-transcriptional gene silencing mechanism and RNA interference. miRNAs suppress post-transcriptional activity, acting on several genes and many of these non-coding nucleotides are related to adipocyte development, lipolysis, insulin sensitivity, glucose uptake (34), and multiple cytokines release (35). It is known that hundreds of miRNAs can regulate up to 30% to 80% of genes encoded in the human genome, which each miRNA can target more than one hundred genes, undertaking gene regulation (36) A single miRNA may have multiple target sites in the 3’UTR region of a specific mRNA (messenger RNA) (36). The biogenesis of the miRNA can be distinguished into a canonical and non-canonical pathway.

**Figure 2:**
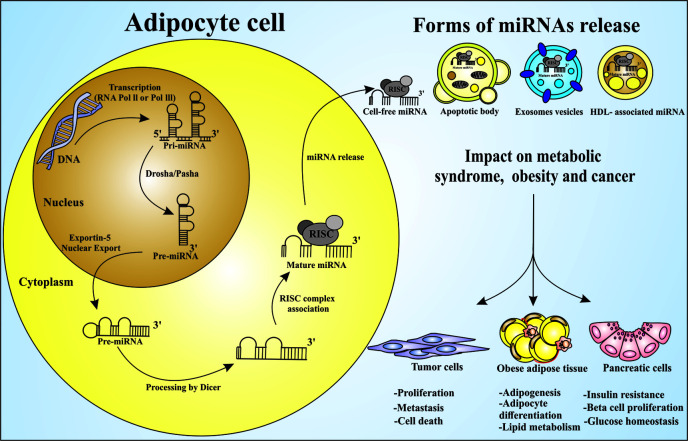
miRNAs processing and releasing by adipocytes and their influence on metabolic syndrome, obesity and cancer. Adipocytes are the main source of circulating miRNAs in mice and human. The process of miRNA maturation begins in the nucleus with the gene transcription by RNA polymerase II or III in pri-miRNA. Following the maturation process, the enzyme DROSHA and its Pasha cofactor leads to the pre-miRNA, which are exported to the cytosol by the Exportin-5 protein, and further processed by Dicer enzyme, leading to the mature miRNA. It is taken into the RISC silencing complex and then become an active post-transcriptional regulator. This may occur by cell free miRNA release, associated with apoptotic body, exosomes, or HDL. miRNAs can act influencing different types of cells. Circulating miRNAs are able to control: (I) the expression of several genes related to adipogenesis, (II) the adipocyte differentiation and lipid metabolism impacting obesity, (III) the expression of membrane proteins responsible for the glucose homeostasis and insulin resistance, (IV) the metabolic diseases development, and (V) the cancer establishment and progression as oncomiR molecules.

The canonical transcriptional pathway begins with the RNA polymerase II/III leading to a long primary miRNA transcript, known as pri-miRNA, which has a 5’ cap and a poly A tail (37). The pri-miRNAs are transcribed and processed into pre-miRNAs by a complex consisting of an RNA-binding protein called DGCR8 (DiGeorge Syndrome Critical Region 8) and a ribonuclease III, Drosha, while DGCR8 recognizes the N6-methyladenylated GGAC and other motifs in the pri-miRNA are recognized by DGCR8. The DROSHA cleaves the duplex of pri-miRNA in the structure of the hairpin, resulting in the pre-miRNA. The pre-miRNA is transported into the cytoplasm by exportin 5 (XPO5)/RanGTP and then processed by Dicer RNase III endonuclease (38). The miRNA biogenesis and the impact of miRNAs on metabolic syndrome, obesity and cancer are summarized in **Figure 2**. The removal of the terminal loop leads to the mature miRNA duplex, which in turn is incorporated into a multimeric complex called RISC, including the Argonaut proteins as the main components, in an ATP-dependent manner.

In the canonical pathway, miRNAs are generated by transcriptional units of protein-coding, while in the non-canonical pathway they are generated from non-protein-coding transcriptional units. Another critical difference between these two pathways is that in a canonical way the intronic miRNAs are Drosha-dependent and then processed with protein-coding transcripts in the nucleus. The pre-miRNA undergoes splicing producing the mature mRNA which directs to protein synthesis. In the non-canonical pathway, small introns of RNA, called mirtrons, are not processed by Drosha (39).

miRNAs show an essential role in gene expression, through mRNA degradation, deadenylation, or inhibiting the protein translational (40). Recent studies have shown that inhibition of Drosha and Dicer enzymes, critical in miRNA biogenesis, corroborated to the blockage of adipocytes differentiation and adipogenesis. Adipocytes are the major site of exosomal miRNAs, acting on cell to cell communication, as biomarkers in several diseases, including type 2 diabetes and cancer (41). The Dicer enzyme processes miRNA in the cytoplasm, a crucial stage of the miRNA biogenesis. The silencing of Dicer enzyme in adipose tissue, in a mice model, exhibited abnormal fat accumulation in the interscapular region and intra-abdominal, whitening of brown adipocytes, lipodystrophy, dyslipidemia, and insulin resistance (42). Lipodystrophy, characterized by lipoatrophy, loss of adipose tissue or abnormal distribution in specific regions anatomically, is a clinical manifestation of metabolic syndrome, as well as increased visceral fat accumulation and dyslipidemia (43).

## The Role of miRNAs in White and Brown Adipose Tissue

Several works have shown the role of miRNAs in the development of metabolic disease, glucose homeostasis, and progression of obesity (44–47). miRNAs can exhibit a crucial impact on adipose tissues (**Table 1**) and influence the phenotype and function of white and brown adipocyte (75). miRNAs can function both as activators and inhibitors of brown adipogenesis and are crucial for adipose tissue plasticity and dynamics of adipocytes differentiation (76, 77). The absence of miRNAs can impact the differentiation of white adipocyte into brown adipocyte dynamics and can be involved in the recruitment and activation of thermogenic adipocytes (75). There is a strong correlation between miRNAs and the regulation of browning of the WAT.

**Table 1:** The dynamics of miRNAs functions in adipose tissue microenvironment.

miRNAs	Tissue	Species	Highlights	References
miRNA-193b/365	BAT and WAT	Mouse	Myogenesis and adipogenesis	(48)
miRNA-196a	sWAT	Mouse/Human	Brown adipogenesis	(49)
miRNA-103	3T3-L1	Mouse	Insulin signaling impairment	(50, 51)
miRNA-133a and miRNA-133b	BAT and sWAT	Mouse	Brown adipogenesis inhibition	(52, 53)
miRNA-27	BAT and sWAT	Mouse/Human	White adipogenesis regulation; higher risk in gastric cancer	(54, 55)
miRNA-26a and miRNA-26b	sWAT	Human	Adipocyte differentiation; insulin signaling activation	(56, 57)
miRNA-145	MSC	Mouse	Insulin stimulated AKT pathway inhibition and tumor suppressor in lung adenocarcinoma	(58, 59)
miRNA-34a	BAT and liver	Mouse/Human	Browning adipogenesis suppression; antitumoral role in breast cancer	(60, 61)
miRNA-455	BAT and WAT	Mouse	Brown adipogenesis activation	(62)
miRNA-221 and miRNA-222	SWAT/white preadipocytes	Human	Vascular Remodeling; epithelial-to mesenchymal transition activation in breast cancer	(63, 64)
miRNA-125b	White preadipocytes	Human	Reduction of mitochondrial biogenesis, higher risk in prostate cancer	(65, 66)
miRNA-320	3T3-L1	Mouse	Insulin sensitivity inhibition	(67)
miRNA-33	Subcutaneous Preadipocytes	Human	Lipid homeostasis regulation	(68)
miRNA-375	3T3-L1, hASCS, pancreatic islets	Mouse/human	Glucose homeostasis regulation	(69)
miRNA-377	3T3-L1	Mouse	Adipogenesis regulation and tumor suppressor through SIRT1 inhibition	(70)
miRNA-21	ADSC	Human	Adipogenic differentiation and oncogenic through a mechanism dependent on PTEN downregulation	(71, 72)
miRNA-155	Brown preadipocytes	Mouse/Human	Adipogenesis, hypoxia activation and, overexpression in lung cancer	(73, 74)

WAT, white adipose tissue; BAT, brown adipose tissue; PTEN, Phosphatase and tensin homolog; SIRT1, sirtuin 1; hASCs, human adipose tissue stem cells; MSC, adipose tissue–derived mesenchymal stem cells; ADSC, adipose tissue–derived stem cells.

Adipose tissue–specific DGCR8 knockout mice presented enlarged, but pale interscapular brown fat with decreased expression of characteristic genes of brown fat and intolerant to cold exposure (78). miR-182 and miR-203 were identified as key regulators of the brown adipocyte development (78). The recruitment and activation of constitutive BAT in humans, as well as the transdifferentiation of visceral and subcutaneous WAT into brown adipocytes appear as a new strategy for obesity treatment (79). BAT associated thermogenesis increases caloric burning, contributing to accelerating weight loss. That highlights the perspectives of these new targets to restore the normality of energy homeostasis and to struggle the progression of obesity by modulating the plasticity of adipose tissue, mainly visceral WAT (80). Since miRNAs are deeply associated with adipocytes differentiation and obesity, circulating miRNAs are very attractive biomarkers and therapeutic tools for obesity prevention and treatment (46).

The miRNA cluster miR-193b and miR-365 may display a regulation of brown fat fate through myogenesis and adipogenesis. Sun and colleagues compared the genome-wide miRNA profile expression of the epididymal WAT, skeletal muscle and interscapular BAT. The WAT samples presented an upregulation of miR-193b and miR-365 during adipogenesis, and a downregulation of up to 30% in BAT of ob/ob mice (metabolic impairment). The authors showed that miR-193b induced myoblasts to differentiate into brown adipocytes, and demonstrated that miR-193b and miR-365 365 can serve as essential regulators for brown fat differentiation, in part by repressing myogenesis (48).

The miR-103 expression could be related to impaired insulin sensitivity and glucose uptake, possibly an attractive pharmacological target in obesity and diabetes, promoting cell adipogenesis by targeting MEF2D and activating AKT/mTOR signaling pathway (81). The miR-103/107 also trigger endoplasmic reticulum (ER) stress-mediated apoptosis by targeting the Wnt3a/β-catenin/ATF6 signaling pathway in preadipocytes impairing adipogenesis (82). Overexpression of miR-103/107 was observed in obese mice and resulted in augmented accumulation of triglycerides and impaired regulation of adipogenic genes, while silencing of miR-103/107, improved glucose homeostasis and insulin sensitivity (51). Caveolin-1 could explain one of the mechanisms discussed for negative regulation regarding insulin sensitivity of miRNA-103. The miR-103 targets caveolin-1 and diminishes the expression of insulin receptors in the caveolae-enriched plasma membrane (51).

miRNAs also display a master role in brown adipogenesis of white fat progenitor cells, as reported for miR-196 in WAT. This miRNA suppresses the transcriptional factor HOXC8, avoiding the repression of its target C/EBPβ (CCAAT/enhancer binding protein β), considered a key regulator of BAT gene (49). Moreover, cold stimulus and β-3 adrenergic activation in white adipocytes can activate miR-196 in WAT from mice (49).

miR-133 is involved in BAT activation. miR-133 directly targets and negatively regulates PRDM16. In addition, inhibition of miR-133 can promote differentiation of precursors from BAT to mature brown adipocytes and increased mitochondrial activity in response to cold exposure (83). The upregulation of PRDM16 is linked to a decrease of miR-133a expression and browning of WAT enhancement (53), suggesting that pharmacological strategies to inhibit miR-133 could be very prominent to promote browning of the WAT. Likewise, miR-155 is enriched in BAT but efficiently inhibits adipogenic differentiation and development of the thermogenic program in brown adipocytes. Moreover, miR-155–null mice exhibit enhanced BAT function, while overexpressing miR-155 in mice induced a diminished browning markers expression, such as UCP1, BAT mass reduction, and a total impairment of BAT function (84).

miR-27 can negatively regulate brown and white adipogenesis both in mice (55, 85, 86) and humans (87, 88). miR-27 is reported to promote adipogenesis of WAT, targeting the 3’UTR region of PPARγ (Peroxisome Proliferator-Activated Receptor gamma) (17). The cold exposure can downregulate miR-27 expression in murine BAT and subcutaneous WAT (55). The miR-27 can also reduce the expression of brown markers genes, such as PRDM16, PPARα, CREB (Cyclic AMP Response Element Binding Protein), and PGC1β (Peroxisome Proliferator-Activated Receptor Gamma Co-activator 1 β). Moreover, the overexpression of miRNA-27 is also related to a higher risk of gastric cancer, promoting a transformation of cancer-associated fibroblasts (54).

miR-26a and miR-26b can trigger adipocytes differentiation, both in human and mice studies. The overexpression of miR-26a and miR-26b promote adipocyte differentiation of human multipotent adipose derived-stem cells (56). miR-26b also modulates insulin-stimulated AKT activation *via* inhibition of its target gene, PTEN (Phosphatase and tensin homolog), and significantly increases insulin sensitivity *via* the PTEN/PI3K/AKT pathway (57). Moreover, overexpression of miR-26b significantly increases the mRNA expression of the adipogenic markers, PPARγ, fatty acid synthase (FAS), CCAAT/enhancer-binding protein alpha (C/EBPα), and lipoprotein lipase (89).

The miR-143, miR-145, and miR-130 also exert a role in adipogenesis. The miR-130 inhibits PPARγ expression (90). The liver analysis from obese mice shows an increased expression of miR-143 and miR-145, also an association of these miRNAs expression and changes of insulin-stimulated AKT signaling pathway. The knockout mice for miR-143 and miR-145 did not develop obesity in insulin resistance conditions (58).

miRNAs are differentially expressed in white and brown adipocytes and may be important in defining the common precursor cell for myocytes and brown adipocytes and thus have distinct roles in energy‐storing versus energy‐dissipating cells. The miR-455 is related to browning of WAT induction, upregulating UCP-1 expression. Adipose-specific miR-455 transgenic mice exhibit brown fat characteristics in subcutaneous white fat, after cold exposure. miR-455 boosts AMPKα1 (AMP-activated kinase) activation through HIF-1α (hypoxia inducible factor 1 α subunit) inhibitor (62). The AMPKα1 contributes to brown adipogenic effect and induces mitochondrial biogenesis (91). The classic myogenic miR-1 and miR-206 are absent in murine white adipocytes and expressed both in brown pre‐ and mature adipocytes, strengthening the concept that brown adipocytes and myocytes can share a similar progenitor cell lineage that specifies energy‐dissipating cells (92).

A higher miR-34a expression was detected in obese mice (60) and on serum analysis from patients with type 2 diabetes (93). The miR-34 suppresses browning of WAT, directly affecting adiposity, adipocyte differentiation, and metabolism. It targets FGFR1 (Fibroblast Growth Factor Receptor 1) and SIRT 1(sirtuin 1) (60). The suppression of SIRT1 (sirtuin) is related to impaired transcriptional regulation of brown and beige markers through deacetylation of PPARGC1-α (60, 61). The lentiviral-mediated knockdown of miR-34a under a high-fat diet have shown a different pattern of perirenal and gonadal WAT depots distribution, and less ill metabolic effects (94).

Microarray analysis of human WAT has revealed distinct miRNA expression that may be dysregulated in patients with obesity and other comorbidities, such as type 2 diabetes mellitus and heart diseases (95). Dysregulation of miR-20, miR-98, miR-197, and miR-331 were common in patients with obesity and type 2 diabetes mellitus (96). The molecular pattern of miRNAs may be distinct in patients with morbid obesity, as seen in a circulating genome-wide miRNA pattern from obese patients before and after bariatric surgery. The results revealed a differentiated profile of miRNA expression in cases of severe obesity. Among these miRNAs the miR-142-3p, miR-140-5p, miR-15a, miR-520c-3p, and miR-423-5p were identified as potential new biomarkers of severe obesity (97).

The overall miRNA screening of white subcutaneous adipocytes performed in obese patients and in lean patients reported an overexpression of miR-143-3p and miR-652-3p in obese patients when compared to lean patients, also detected a higher chemokine CCL2 production and lipolysis from necrotic adipocytes from obese WAT, demonstrating an inflammatory response triggered by these miRNAs overexpression. The expression of these miRNAs has been reported to increase insulin-stimulated lipogenesis, affecting genes at transcriptional and post-transcriptional levels of insulin signaling (98).

The dysregulation of miRNAs expression in white and BATs can be a valuable target and biomarker in diseases associated with adipose tissue dysfunction, such as metabolic syndrome and cancer (99, 100).

## The Function of Adipose Tissue miRNAs in Metabolic Syndrome

Metabolic syndrome is a cluster of cardiometabolic risk factors characterized with obesity, hyperglycemia, dyslipidemia, hypertension, atherogenic, prothrombotic, and proinflammatory states, NAFLD, often associated with a condition of insulin resistance (101, 102). Adipose tissue miRNAs have direct impact on metabolically important organs, modifying cell physiology and acting as key mediators of metabolic syndrome features. miRNAs can regulate multiple pathways including insulin signaling, meta-inflammation, adipogenesis, lipid metabolism, and food consumption (103). The miRNAs are strongly related to immune response dysfunctions in metabolic syndrome, as in macrophages polarization (104), adipogenesis regulators and mediators of the fatty acids metabolism, also contributing to adipocyte hypertrophy in obesity, hepatic steatosis, and lipid homeostasis disorders, insulin signaling pathways, β-pancreatic cells mass, and endocrine function (105).

The first evidence highlighting miRNAs role in metabolic syndrome was described in the studies using Drosophila adipocytes, demonstrating that miR-14 had a suppressive effect on fat metabolism through the MAPK and p38 pathways in this model (99). The screening of several miRNAs demonstrated correlation between obesity and metabolic syndrome, specially linked to insulin resistance and regulation of β-pancreatic cells. The beta-pancreatic cell death can trigger pancreatic dysfunction and promote type 2 diabetes. Studies have reported the overexpression of the miR-200 family in pancreatic islets. The analysis of diabetic mice showed a negative regulation of miR-200 in anti-apoptosis pathways, thus favoring beta-pancreatic cell death (106). The overexpression of miR-7 in beta cells from mice showed an impairment of insulin granule exocytosis (107).

Meta-inflammation is a common feature of metabolic syndrome, and miRNAs can actively regulate this process, by altering macrophages phenotype and modulating the inflammatory molecules secretion within the adipose tissue. Studies reported the ablation of miR-223 in mice undergoing a high-fat diet, resulted in increased M1 macrophages infiltration, as well, higher pro-inflammatory cytokines secretion, such as IL-6, IL-12, TNF-α, and IL-1β (108). This meta-inflammation is also closely related to changes in the proliferative pathways, as seen for miR-21 dysfunction in obesity, affecting angiogenesis and cell growth (103). These miRNAs-mediated alterations detected in metabolic syndrome, such as exacerbated meta-inflammation, enhanced c proliferative and a pro-angiogenic microenvironment in WAT, could partially explain the higher predisposition to several types of cancer observed in people with obesity.

The miRNAs dysfunction in obesity and metabolic syndrome was detected on the FXR/SHP signaling pathway. Fat metabolism and cholesterol homeostasis are one of the axes that can be unbalanced in metabolic syndrome. The miR-33 is related to cholesterol trafficking *in vitro* and HDL synthesis also regulates cholesterol transport and lipid homeostasis (68, 109). The FXR/SHP can inhibit miR-34a expression (110). The Farnesoid X Receptor (FXR) is a nuclear receptor, controlling the regulation of genes expression related to fatty acids metabolism, cholesterol levels, through inhibition of cytochrome P450 7A1, which is necessary for hepatic bile acids synthesis. The effect of miR-34a inhibition leads to an upregulation of SIRT1 and dysregulation of FXR/SHP pathway in diet- induced obese mice (111). The miR-34a can also reduce the NAD+ (Nicotinamide Adenine Dinucleotide) and SIRT1 activity, targeting NAMPT (Nicotinamide Phosphoribosyltransferase), a key enzyme for NAD+ synthesis. The overexpression of miR-34a increases mRNA degradation of NAMPT, resulting in decreased levels of NAMPT/NAD+. The dysregulation on this pathway alters PGC-1α (Peroxisome Proliferator-Activated Receptor Gamma Coactivator 1-alpha), SREBP-1c (Sterol Regulatory Element-Binding Transcription Factor 1), and NF-κB, enhancing obesity. The antagonism of miR-34a reduces hepatic steatosis, glucose intolerance, and inflammation. The inhibition of miR-34a represents a potential therapeutic target against obesity and metabolic syndrome (112).

The miR-30c is related to hMADS (human multipotent adipose-derived stem cells) and promotes adipogenesis and accumulation of triglycerides. The miR-30c targets PAI-1 (Plasminogen Activator Inhibitor-1) and ALK2 (Activin-like kinase receptor 2), which are downregulated by miR-30c in adipogenesis. The correlation of miR-30c and PAI-1 was observed in WAT from obese mice, suggesting an endocrine regulation role of these molecules during obesity (113).

Concerning the insulin regulation by miRNAs, recent reports suggest post-transcriptional mediated by IGF-1 (insulin-like growth factor 1) and INSR (insulin receptor) activation (114), GLUT4 protein expression (115), pancreatic beta cells proliferation, and differentiation (116, 117). The inhibition of miRNA-320 resulted in increased insulin sensitivity, improved AKT phosphorylation and GLUT4 expression (67, 118). The miRNA-93 expression was inversely correlated with GLUT4 levels in adipose tissue from non-obese insulin-resistant patients and its overexpression in adipocytes downregulated GLUT4 expression (119).

miRNA-375 is one of the most abundant miRNAs in pancreatic beta cells and overexpression of miR-375 can suppress glucose-induced insulin secretion, and conversely, inhibition of endogenous miR-375 function can enhance insulin secretion (110). This process is mediated by myotrophin and PDK1 (Phosphoinositide-dependent kinase-1), a key molecule in PI 3-kinase signaling (120). The silencing of miR-375 in leptin-deficient diabetic mice resulted in hyperglycemia, increased glucagon concentration, and gluconeogenesis. In addition, miRNA-375 knockout mice presented an altered pancreatic β cell mass, resulting in severe diabetes (69). The miR-375 low levels in pancreatic beta cells are associated with pancreatic cancer progression, metastasis, and angiogenesis (121).

Indeed, growing data have indicated a strong association of metabolic syndrome or its components with cancer development and cancer-related mortality (122–127). It is possible for the association between metabolic syndrome and cancer to be mediated by the dysfunctional adipose tissue, exacerbated meta-inflammation, coexistence of obesity status, and insulin resistance as the main factors. Importantly, adipose tissue miRNAs could be the one of the key molecules orchestrating this crosstalk linking metabolic syndrome and cancer.

## The Role of Adipose Tissue miRNAs in Cancer

Hanahan e Weinberg described the hallmarks of cancer, providing new insights of carcinogenesis establishment, such as self-sufficiency in growth signals, evading apoptosis, insensitivity to anti-growth signals, sustained angiogenesis, limitless replicative potential, tissue invasion and metastasis, immune response evasion and metabolic reprogramming, genetic instability, mutations, and tumor-promoting inflammation (128, 129). The high plasticity of the adipose tissue and its inflammatory content can directly regulate the tumor microenvironment, defining cancer establishment and progression (12). The crosstalk between adipocytes and other components of the adipose tissue, such as immune cells, can be crucial to determine the cancer cells fate (12). Adipose tissue–derived circulating miRNAs, considered novel adipokines (130), can affect important signaling pathways, directly impacting the carcinogenesis pillars.

Several studies reported a higher risk of cancer in patients with obesity. There are at least 13 types of cancer which this correlation is already described, such as esophagus (131), gastric (132), colon and rectum (133), liver (134), prostate (135), ovary (136), kidney (137), meningioma (138), thyroid (139), and multiple myeloma (140). miRNA expression in adipose tissue from people with obesity can play a crucial role in tumor progression, cancer growth and metastasis. miRNAs mediate the crosstalk of adipocytes and macrophages, modulating the inflammatory and angiogenic microenvironment (141, 142). miRNAs are indeed dysregulated in most, if not all, cancers (143). However, the function of several miRNAs in cancer is very controversial in the literature (144). Many reports are demonstrating unique miRNAs acting both as miRNAs oncogenic (oncomiR) and tumor-suppressive. Specific miRNAs can simultaneously trigger anti-cancer effects by suppressing oncogenic mRNAs, and pro-tumoral effects by suppressing tumor suppressive mRNAs. A balance between these two signaling pathways will determine whether a miRNA is an oncomiR or a tumor-suppressive molecule (144).

The miRNA-377 is considered the master adipogenesis regulator under stress stimulus in WAT (70). Studies reported miRNA-377 targeting SIRT1 and reducing its expression in diet obese mice, causing inflammation and insulin resistance in 3T3-L1 cells (70). SIRT1 exerts a role in tumor initiation, progression, and angiogenic pathways (145), as depicted in murine hepatocellular carcinoma (146), highlighting the potential antitumoral role of this miRNA, through a SIRT pathway. SIRT1 is also correlated with inflammation and insulin resistance, which contributes to the development of a pro-tumoral environment, increasing the risk of ROS (reactive oxygen species) production, thus leading to DNA damage and mutations, genome instability, one of the hallmarks of carcinogenesis (147).

The miRNA-34 family includes the miRNA-34a, miRNA-34b, and miRNA-34c clusters, found in WAT. The tumoral suppressor p53 is a direct transcriptional target of miR-34. The p53 is activated under cellular stresses and promotes cell cycle arrest, senescence, and apoptosis (148). Recent data also reports the role of p53 in immune response, metabolism, and inflammation (149). The miRNA-34 inhibits the proliferation of breast cancer cells through a mechanism of SIRT1 and Bcl-2 downregulation (61), and thus miR-34 seems to have an anti-tumor effect (150).

The cluster of miR-221 and miR-222 exerts a role in vascular remodeling in atherogenic processes, inhibiting the angiogenic recruitment of endothelial cells, thus facilitating the atherogenic calcification (63). These miRNAs are also related to human breast cancer development, controlling the epithelial-to-mesenchymal transition, downstream the oncogenic RAS-RAF-MEK pathway (64). The miR-223 controls macrophage polarization preventing mice from inflammation induced by diet and insulin resistance. The anti-inflammatory mechanism described for miR-223 is the suppression of classical proinflammatory M1 macrophages response in adipose tissue (108).

The miR-125b controls mitochondria integrity and is related to metabolic adaptations of monocytes, driving inflammation, and apoptosis (65). The miR-100 was identified as an important factor maintaining the M2 phenotype of tumor associated adipose tissue macrophages. The miR-100 targets the mTOR pathway and was also observed in some studies a higher expression in tumor-associated macrophages isolated from mouse breast tumor tissue. The therapeutic strategy of combining miR-100 inhibition with cisplatin could suppress mouse lung metastasis (151).

The miR-193b/126/26a are up-regulators of adiponectin secretion in human white adipocytes, modifying the expression of adiponectin regulators genes, such as the nuclear receptor-interacting protein 1 and nuclear transcription factor Yα (152). Adiponectin augments adipogenesis and prevent ectopic lipid accumulation as liver steatosis, exerting a protective effect against insulin resistance and cardiovascular diseases (153). Meta-analysis studies demonstrated that low levels of adiponectin are correlated to a higher risk of postmenopausal breast cancer (154) and increased adiponectin serum levels confered a protective effect on endometrial cancer (155).

Adipogenesis and immunological recruitment can alter the tumor microenvironment. The miR-183 targets low-density lipoprotein receptor-related protein in 3T3-L1 cells, promoting differentiation and adipogenesis by interfering with the Wnt/β catenin pathway (156) and tumor-derived TGF-β promotes miR-183 expression, silencing NK cells, through a mechanism mediated by DAP12 suppression. The DAP12 downregulation facilitates the cancer establishment, avoiding NK cells to recognize and eliminate the tumor cells, maintaining cancer cells survival and spread, in a pathway dependent on miR-183, suggesting a pro-tumoral role for this miRNA (157).

The miR-21 contributes with adipogenic differentiation of human adipose tissue–derived mesenchymal stem cells, through TGF-β signaling (71) and is also associated with cancer. The overexpression of miR-21 was detected in pancreatic, colon, breast, and glioblastoma tumors (158). Studies reported the overexpression of miR-21 in colorectal cancer with advanced metastasis stage. miR-21 is associated with anti-apoptotic effects, promoting cellular proliferation by targeting tumor suppressors, such as PTEN. Moreover, this event was inversely correlated with miR-21 expression, as summarized in **Figure 1** (72). Some miRNAs act inhibiting tumor progression, leading to oncogenes mRNA degradation or repression of the oncogenic mRNA translation (159). The miR-15a/miR-16-1 cluster interacts with Bcl-2 mRNA, resulting in leukemic cell apoptosis (160).

The secretion of miR-31 from adipose tissue–derived stem cells is associated with endothelial cell migration and angiogenesis (161). Furthermore, Factor Inhibiting HIF-1α (FIH), an anti-angiogenic gene is one of the targets of miR-31, indicating a possible correlation between hypoxia, adipose tissue dysfunction, and tumoral signaling pathways (161). Besides angiogenic pathways facilitating the tumor cells spread, the mitochondrial biogenesis and oxygen consumption can also be associated with tumor growth (162). miRNAs can induce mitochondrial metabolic alterations and a shift from oxidative to glycolytic metabolism, by targeting components involved in electron transport chain/oxidative phosphorylation (OXPHOS) and in tricarboxylic acid cycle, thus contributing to Warburg effect and cancer progression (163, 164). miR-126 can affect mitochondrial function and mitochondrial metabolism in cancer cells, leading to tumor suppression of cancer cells by inducing tumor cells metabolic reprogramming (165). Obesity can reduce the miR-126 expression (166), and this miRNA is often lost in several types of cancer (167).

In both humans and mice, the miR-125b expression is higher in WAT than in BAT, especially during obesity. It has been shown that miR-125b-5p can play an important role in the repression of brite adipocyte function by modulating oxygen consumption and mitochondrial gene expression (66). The higher expression of miR-125b was shown to be related to prostate cancer risk, in a mechanism associated with androgenic receptor stimulus, also promoting tumor growth by modulating the cell cycle, apoptosis, and cell migration (168). The role of miR-125b in stem-cells hematopoiesis, as well as the activation and expression of this miRNA in human leukemia, shows its function as an oncomiR (168). However, the role of miR-125 in cancer is very controversial, since miR-125b can act not only as an oncomiR in the vast majority of hematologic malignancies, but also as a tumor suppressor in many solid tumors (169, 170).

The miRNA-130 family, including miRNA-130a and miRNA-130b, is deeply related to cancer progression (109, 171). It has been demonstrated the correlation of miR-130a and miR-130b expression in preadipocytes isolated from abdominal subcutaneous human adipose tissue and PPARγ repression, inhibiting adipogenesis (90). Likewise, PPAR γ activation could be protective against gastric cancer (172). Increased levels of miR-130b were found in advanced tumor stages (III–IV) of colorectal cancer patients (109). The pharmacological inhibition of miR-130 family molecules by seed-targeting with an 8-mer tiny locked nucleic acid (LNA) inhibited bladder cancer cell growth, migration, and invasion by repressing stress fiber formation (173). Overall, miRNA-130 is highly expressed in patients with different types of cancer and is correlated with severe tumors and poor cancer prognosis (174).

The miR-193b-365 cluster is defined as a brown fat-enriched cluster, already depicted in lineage determination of brown adipocytes, and brown adipocyte adipogenesis in mice studies (105). The miR-193 has shown an anti-tumoral effect in breast cancer, by decreasing cell proliferation and migration (175). Besides, miR-193a-3p suppressed cancer gastric growth and motility (176). The miR-365 expression was downregulated in glioblastoma (177) and inhibited cell proliferation of glioma and malignant melanoma (177–179).

Studies reported the silencing of miR-145 associated with increased liver inflammation in mice. Likewise, the lentivirus-mediated miR-145 inhibited macrophage infiltration, ameliorated glucose metabolism, and weight loss (180). This target has been characterized as a tumor-suppressor miRNA, impacting cell growth (59), leading to Myc proto-oncogene downregulation (181). The miR-145 suppressed gastric cancer cell proliferation through cell cycle arrest and apoptosis (182).

Another crucial miRNA related to BAT, is the miRNA-155. The miRNA-155 acts in the adipogenic transcription factor CCAAT/enhancer-binding protein β (84) and HIF-1α (73). Studies demonstrated a higher expression of miR-155 in mice intestinal tissue under hypoxia stimulus (73). Inflammation upregulates miRNA-155 as seen in biopsies taken from patients with obesity, and TNFα- treated adipocytes, contributing to impair adipocytes function, as summarized in **Figure 1** (183). It was also described the correlation of miR-155 expression in lung cancer and hypoxia, which impacts tumor microenvironment and cancer progression (74). The miR-155 can be involved in the chemotherapy resistance, and this could be due to hypoxia and inflammation signaling pathways (184).

According to the miRNA target, the miRNA-based therapies can follow two opposite directions, acting as tumor suppressor or oncogenic molecules. The therapeutic strategy can be based on miRNA antagonist molecules such as synthetic oligonucleotides (antagomiRs), locked nucleic acids (LNA), a class of nucleic acid analogs, capable of forming base pairs with complementary nucleosides, demonstrating high affinity and resistant nucleases degradation (185), silencing the oncogenic miRNA and finally the miRNA mimetics in the tumor, restoring the protective action of the miRNAs. AntagomiRs are oligonucleotides developed to silence specific miRNA. Studies have reported that intravenous administration of antagomiRs in mice accomplished great results, with high specificity and low toxicity (186).

The MRX34 drug, the liposomal formulation of a synthetic miRNA-34a followed to phase 1 clinical trial. It was administered intravenously in patients under advanced tumor stage and refractory to standard chemotherapy. The drug strategy was based on mimic miRNA therapy, to restore tumor suppressor miRNAs levels, in solid cancers, such as liver and spleen (187). However, due to serious adverse events, including immune reactions, the clinical trials did not proceed to phase 2. Although advances to clinical phase 3 have not been described, new candidates, such as the drug RGLS5579, targeting miR-10b in glioblastoma and inhibitors of miR-155 and miR-21 in the treatment of hematological, breast and lung tumors have been developed (188). In addition, LNA-anti-miR-21 for targeting miR-21 expression to treat colon adenocarcinoma has been investigated (189).

Remarkably, miRNAs are related to all hallmarks of cancer, including uncontrolled cell proliferation and growth capacity, cell death evasion, tumor-promoting inflammation signals, antigrowth signals, improved pro-angiogenic function, metastatic potential, energy metabolism reprogramming, immune response evasion, and genomic instability. Overall, several epigenetic and genetic modifications may occur in cancer cells and lead to the gain of function of oncomiRs and loss of function of tumor suppressor miRNAs. It is important to note that several miRNAs can act both as oncomiR and tumor suppressor molecules. Therapies based on miRNA must also consider that cancer cells present an altered miRNAs expression, and tumor phenotype can be changed by the modulation of the miRNA expression. Therefore, a careful design and approach, is essential to use miRNAs as very prominent therapeutic tool against cancer.

## Conclusion

Obesity can often lead to metabolic impairment, triggering inflammation, and contributing to disorders related to adipogenesis and lipid metabolism. Adipose tissue–derived miRNAs exert a crucial role in adipocyte differentiation, adipogenesis, and hormone homeostasis being deeply related to the establishment of metabolic syndrome, obesity and cancer. miRNAs can act as oncogenes and tumor suppressor factors, depending on its expression and mRNA target, demonstrating a defying way to pharmacological strategies, according to the tumor microenvironment and the impaired signaling pathways. The ever growing data showing the involvement of miRNAs in cancer progression and invasion is supporting the arising use of miRNAs for clinical assessment of cancer aggressiveness and tumor biomarkers, as well as turning miRNAs in promising molecules for therapeutic anti-cancer approaches. miRNAs can deeply change important physiologic characteristics of white and BATs, impacting browning of the WAT, modulating the inflammatory response, and therefore influencing directly the obesity and metabolic syndrome status, and thus cancer predisposition. Moreover, adipose tissue is considered as the major source of circulating miRNAs, acting both in paracrine and endocrine signaling, modulating tumor microenvironment, affecting cancer establishment and progression. Therefore, adipose tissue–derived miRNAs may represent a promising therapeutic target against metabolic syndrome, obesity, and cancer.

## Author Contributions

All authors contributed to the article and approved the submitted version.

## Conflict of Interest

The authors declare that the research was conducted in the absence of any commercial or financial relationships that could be construed as a potential conflict of interest.
